# Plasma Lectin Pathway Complement Proteins in Patients With COVID-19 and Renal Disease

**DOI:** 10.3389/fimmu.2021.671052

**Published:** 2021-04-29

**Authors:** Nicholas R. Medjeral-Thomas, Anne Troldborg, Annette G. Hansen, Jack Gisby, Candice L. Clarke, Maria Prendecki, Stephen P. McAdoo, Eleanor Sandhu, Liz Lightstone, David C. Thomas, Michelle Willicombe, Marina Botto, James E. Peters, Matthew C. Pickering, Steffen Thiel

**Affiliations:** ^1^ Centre for Inflammatory Disease, Imperial College London, London, United Kingdom; ^2^ Renal and Transplant Centre, Imperial College Healthcare National Health Service (NHS) Trust, London, United Kingdom; ^3^ Department of Biomedicine, Aarhus University, Aarhus, Denmark; ^4^ Department of Rheumatology, Aarhus University Hospital, Aarhus, Denmark

**Keywords:** COVID-19, coronavirus, lectin, complement, chronic kidney disease

## Abstract

We do not understand why non-white ethnicity and chronic kidney disease increase susceptibility to COVID-19. The lectin pathway of complement activation is a key contributor to innate immunity and inflammation. Concentrations of plasma lectin pathway proteins influence pathway activity and vary with ethnicity. We measured circulating lectin proteins in a multi-ethnic cohort of chronic kidney disease patients with and without COVID19 infection to determine if lectin pathway activation was contributing to COVID19 severity. We measured 11 lectin proteins in serial samples from a cohort of 33 patients with chronic kidney impairment and COVID19. Controls were single plasma samples from 32 patients on dialysis and 32 healthy individuals. We demonstrated multiple associations between recognition molecules and associated proteases of the lectin pathway and COVID-19, including COVID-19 severity. Some of these associations were unique to patients of Asian and White ethnicity. Our novel findings demonstrate that COVID19 infection alters the concentration of plasma lectin proteins and some of these changes were linked to ethnicity. This suggests a role for the lectin pathway in the host response to COVID-19 and suggest that variability within this pathway may contribute to ethnicity-associated differences in susceptibility to severe COVID-19.

## Introduction

Infection with the coronavirus COVID-19 causes a wide range of clinical manifestations from asymptomatic infection to severe respiratory failure and death. The pathogenic mechanisms that determine COVID-19 severity have not been identified. In particular, risk factors that influence individual susceptibility to severe COVID-19, such as the increased risk of COVID-19 death associated with chronic kidney impairment and non-European ancestry, are poorly understood (1–3).

The lectin pathway of complement activation is an important component of innate immunity and contributes to inflammation induced thrombosis, which is a feature of severe COVID-19 (4–6). Upon activation, bioactive forms of complement factors are generated and raised circulating C3a, C5a and C5b9, and increased C5a receptor (type 1) expression associate with severe COVID-19 (7–10). The lectin pathway can trigger these markers of down-stream C3 convertase and terminal pathway activity. Lectin pathway activity is determined by circulating levels of the lectin pattern recognition molecules (PRMs). These are M-, L-, and H-ficolin (also known as ficolin-1, -2 and -3 respectively), mannose-binding lectin (MBL), collectin liver-1 (CL-L1) and collectin kidney-1 (CL-K1). These PRMs circulate in complex with proteases, which include MBL-associated serine protease (MASP)-1, -2 and -3, and non-protease MBL associated proteins, MAp19 and MAp44 (11, 12). Following interaction with ligand, PRM/MASP complexes can cleave complement C4 and C2 proteins resulting in C3 convertase formation and activation of C3, with subsequent activation of the rest of the complement system. Genetic polymorphisms and ethnicity influence the concentration of lectin pathway proteins (13). For example, circulating MBL levels are determined by *MBL2* gene and promoter region polymorphisms, which are present at different frequencies in different ethnic groups worldwide (13, 14). Increased H-ficolin and decreased MBL and MASP-3 levels have been demonstrated in serial plasma samples from patients with sepsis (15, 16). In contrast, no significant differences have been detected in lectin pathway protein levels between young and old healthy adults (17). The potential for lectin pathway PRMs to interact with coronavirus is demonstrated by *in vitro* binding of MBL to SARS-CoV proteins (18).

To date, the only peer-reviewed research of the lectin pathway in COVID-19 are two studies of circulating MBL levels in European-ancestry patients (8, 19). One study detected higher median plasma MBL levels in critically ill COVID-19 patients than healthy controls and associations between MBL levels and pathway activity with thromboembolism (19). However, MBL levels did not associate with survival, the need for mechanical ventilation or acute kidney injury (19). The second study, identified increased C4d and soluble C5b9 (sC5b9) in plasma samples from COVID-19 patients with respiratory failure, but did not demonstrate associations between MBL concentration and COVID-19 severity (8).

To determine whether the lectin pathway contributes to COVID-19 pathogenesis, we measured the plasma concentration of 11 lectin complement proteins and C3dg, a marker of C3 activation, in a population of patients with severe kidney impairment, the majority of whom were of non-European ancestry. The study population provided unique opportunities to understand COVID-19 pathogenesis. Kidney impairment alone is a risk factor for severe COVID-19 (1, 3) and individuals with kidney impairment often have other risk factors for severe COVID-19 such as diabetes, cardiovascular disease and increased age (20). Also, the requirement for individuals with kidney failure to attend for regular dialysis provided a unique opportunity to collect serial samples from patients with mild as well as severe COVID-19 symptoms who would otherwise have self-isolated and recovered in the community.

## Materials and Methods

We screened all patients for symptoms and pyrexia at haemodialysis, clinic or emergency hospital attendance and tested individuals with SARS-CoV-2 nasopharyngeal PCR swab^33^. Study participants provided written informed consent and were enrolled at screening in The Impact of COVID-19 on Renal and Immunosuppressed Patients study (IRAS ID 282077). The study was approved by the Health Research Authority, Research Ethics Committee (reference: 20/WA/0123) and conducted in accordance with Declaration of Helsinki principles.

**Figure 1:**
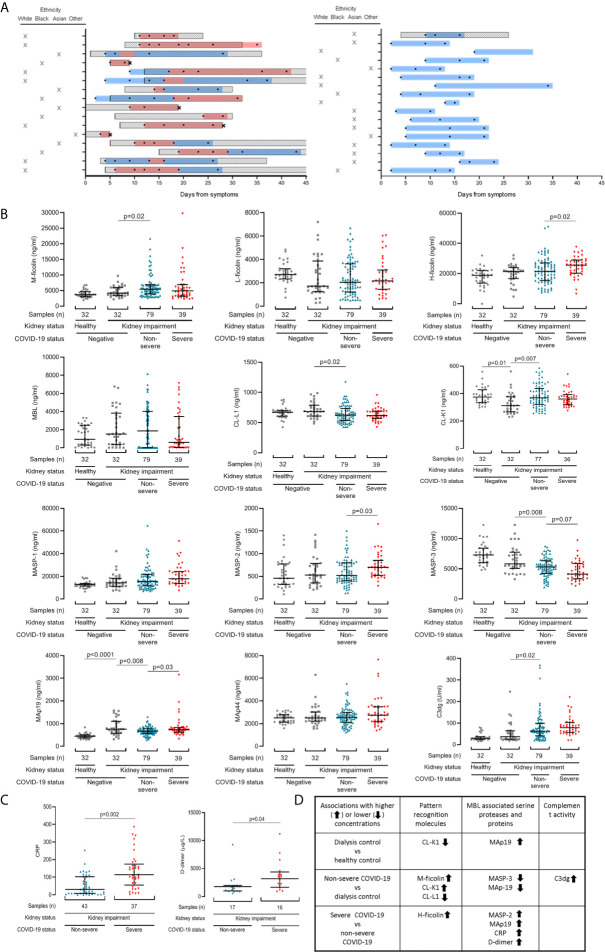
Lectin pathway protein levels associate with COVID-19 severity in patients with chronic kidney impairment. **(A)** Schematic depicting blood sample collection in relation to symptom onset and disease severity. Each row represents one patient. Patients with severe COVID-19 infection are in the left panel, and those with non-severe COVID-19 infection in the right panel. The Y-axis also shows patient ethnicity. Boxed diagonal black lines represent hospital admissions. Two patients (C37 and C16) were hospitalised before developing COVID-19 symptoms. Sampling times are shown with black circles. Black crosses mark patient deaths. Coloured bars show when patients met criteria for severe (red) and non-severe (blue) COVID-19. **(B)** Lectin protein levels in 118 samples from 33 patients with COVID-19. 39 samples were from patients with severe (red triangles) and 79 samples were from patients with non-severe (blue triangles) COVID-19 at sampling. Controls are 32 dialysis patients without COVID-19 (dialysis control cohort, grey squares) and 32 healthy individuals (healthy control cohort, grey circles). **(C)** CRP and d-dimer levels in patients with kidney disease and COVID-19. Lines and whiskers show the median and interquartile values. Differences between cohorts were calculated with a mixed model for repeated measures and adjusted for multiple comparisons as described in the methods. **(D)** Summary of significant associations identified.

We diagnosed COVID-19 from the date of first positive SARS-CoV-2 PCR swab. Blood sampling commenced as soon as feasible after COVID-19 diagnosis. Our research question was formulated and samples collected and processed during the March 2020 COVID-19 pandemic wave in the United Kingdom (UK) when both clinical and research resources were limited. Consequently, we collected samples at pragmatic intervals, but not strictly standardised time points after COVID-19 diagnosis. Additionally, we did not use power calculations to pre-plan the study population needed. We collected 118 serial samples from 33 patients with chronic kidney impairment and COVID-19 (**Figure 1A** and **Supplementary Table 1**) and one sample from each of the 32 dialysis and 32 healthy control individuals. Serial blood samples were taken at the start of haemodialysis sessions and at least 48 hours apart. Of the 118 COVID-19 samples, 80 were collected coincidentally with clinical samples for CRP, 79 for white cell count, 75 for white cell differential cell counts, 40 were coincidental with troponin and ferritin and 33 were coincidental with D-dimer measurements.

We measured concentrations of 11 lectin complement proteins and C3dg. Blood was collected in EDTA tubes and centrifuged to obtain plasma, and stored at –80°C. Sample processing was performed within 4 hours of venepuncture. With the exception of L-ficolin, lectin complement pathway proteins were analysed in EDTA plasma with time-resolved immunofluorometric assay at Aarhus University, Denmark, as previously described (15, 21). Briefly, plasma was thawed, diluted in assay buffers and added to microtiter wells coated with relevant capture antibodies, mannan (for MBL) or acetylated bovine serum albumin (for H-ficolin). All samples were tested in duplicate. Each microtiter plate contained three quality controls, and the intra- and inter-assay coefficients of variation were below 15% for all assays. In-house biotinylated antibodies, europium-labelled streptavidin (PerkinElmer) and enhancement solution (Ampliqon, Denmark) were added in successive steps with triple washing in between, and the europium was detected with a fluorometer performing time-resolved fluorometry. L-ficolin was measured using a commercial enzyme-linked immunosorbent assay (ELISA) (Hycult Biotech, #HK336-02) as instructed by the manufacturer. For the C3dg assay, native C3 was precipitated, plasma was centrifuged and the supernatant was used for analysis at Aarhus University, Denmark, as described previously (22).

Clinical data were collected from electronic medical records, anonymised and stored on secure computer networks at Imperial College Healthcare Trust. We defined COVID-19 severity based on World Health Organisation (WHO) classifications (WHO clinical management of COVID-19: Interim guidance 27 May 2020) adapted for clinical data availability. Mild was defined as COVID-19 symptoms but no evidence of pneumonia and no hypoxia. Moderate was defined as symptoms of pneumonia but peripheral oxygen saturations (SaO2) greater than 92% on air or an oxygen requirement no greater than 4L per minute. Severe was defined as SaO2 less than 92% on air, respiratory rate more than 30 per minute, or oxygen requirement more than 4L per minute. Critical was defined as organ dysfunction, signs of systemic shock or the need for high dependency or intensive care support, for example for non-invasive ventilation or intubation). Severity scores were charted throughout a patient’s illness, including at each sampling point. For some analyses, we combined mild and moderate COVID-19 as ‘non-severe’, and severe and critical as ‘severe’.

### Statistics

Statistical analyses were performed using R Statistical Software and Graphpad Prism 8.0. Protein concentrations were displayed as median with interquartile range (IQR). Differences in clinical characteristics were calculated with the Mann-Whitney U test for continuous and Fisher Exact tests for categorical data. Because repeated measures ANOVA cannot handle missing values, and we had different numbers of samples in each cohort, we analysed differences in lectin protein levels from all available samples by fitting a mixed model in GraphPad Prism 8.0. This mixed model uses a compound symmetry covariance matrix and is fitted using Restricted Maximum Likelihood (REML). We adjusted the data for non-sphericity with the Geisser-Greenhouse correction. Differences between first sample lectin pathway concentrations were calculated with Kruskall-Wallis tests, follow-up comparison of the mean rank of every column, and adjustment of P values for multiple comparisons. We calculated correlations by applying Pearson’s tests to log-transformed data that did not include repeat measures, and linear mixed models with a repeated measures correlation technique (rmcorr) to data from COVID-19 cohorts (23). We adjusted p-values for multiple comparisons using the method of Benjamini and Hochberg with a false discovery rate (Q) of 5% (24).

## Results

We enrolled 33 chronic kidney disease (CKD) patients with COVID-19. 36% (12 of 33) were of Asian and 21% (7 of 33) of Black ethnicity. The controls were 32 haemodialysis patients without COVID-19 (dialysis controls) and 32 healthy volunteers with neither kidney disease nor COVID-19 (healthy controls). Co-morbidity was similar between the dialysis control and COVID-19 cohorts (**Table 1**). Our COVID-19 patient population had a median age of 72 years (range 28-88 years), which was significantly older than dialysis control (62 years, p=0.01) and healthy control (49 years, p<0.0001) cohorts (**Table 1**). We did not consider this to be a limitation because lectin protein levels do not differ significantly between older and younger adults (17). The mean estimated glomerular filtration rates (eGFR) at presentation of the patients not established on maintenance dialysis were 11ml/min/1.73m2 in the CKD patients and 23 ml/min/1.73m2 (range 12-36ml/min/1.73m2) in the kidney transplant recipients.

**Table 1:** Characteristics of COVID-19 and control cohorts.

		COVID-19	Dialysis controls	Healthy controls	Severe COVID-19	Non-severe COVID-19	Difference	95% Cl	p
	Number	33	32	32	16	17			
	Age, years.	72 (range 28-88)	62 (range19-86) *				10	2-15	0.02
				48 (range 28-63) *			24	16-28	0.0001
					64 (28-88)	72 (40-84)			
	Male	22 (67)	19 (59)	17(53)	11 (69)	11 (65)			
Ethnicity	BAME	22 (67)	24 (75)	20 (63)	9 (56)	13 (76)			
	Black	7 (21)	3 (9)	6 (19)	4 (25)	3 (18)			
	Asian	12 (36)	14 (44)	14 (44)	4 (25)	8 (47)			
	White	11 (33)	8 (25)	12 (37)	7 (44)	4 (24)			
	Other	3 (9)	7 (22)	0 (0)	1(6)	2 (12)			
Renal status	Haemodialysis	27 (82)	32 (100)		11 (69)	16 (94)			
	Transplant recipient	3 (9)	0 (0)		2 (13)	1(6)			
	Peritoneal dialysis	1(3)	0 (0)		1(6)	0(0)			
	Chronic kidney disease	2 (6)	0 (0)		2 (13)	0 (0)			
Kidney disease	Diabetic nephropathy	13 (39)	13 (41)		7 (44)	6 (35)			
	Hypertension	3 (9)	0 (0)		1(6)	2 (12)			
	Glomerulonephritis	4 (12)	8 (25)		1(6)	3 (18)			
	Genetic	2 (6)	1(3)		1(6)	1(6)			
	Unknown	5 (15)	9 (28)		3 (19)	2 (12)			
	Other	6 (18)	1(3)		3 (19)	3 (18)			
Co-morbidities	lschaemic heart disease	17 (52)	15 (47)		7 (44)	10 (59)			
	Current smoking	0 (0)	2 (6)		0 (0)	0(0)			
	Ex-smoker	22 (67)	24 (75)		11 (69)	11 (65)			
	Type 2 diabetes mellitus	15 (45)	15 (47)		8 (50)	7 (41)			
	Antihypertensive medications	28 (85)	23 (72)		13 (81)	15 (88)			
	Current immuno suppression	8 (24)	2 (6)		4 (25)	4 (24)			
	Chronic obstructive pulmcnary disease	2 (6)	1(3)		1(6)	1(6)			
COVID-19 progression	Required hospitalisation	17 (52)			16 (100)	1(6)**			<0.0001
	Died from COVID-19	4 (12)			4 (25)	0 (0)**			0.04
COVID-19 clinical biomarker at diagnostic swab	C-reactive protein. NR<5mg/L	60 (IQR 19-114)			91(IQR 41-153)	30 (IQR 7-92) **	61	5 to 101	0.03
	0-dimer.NR <500 ng/ml	1857 (IQR 1152-2899)			1887 (IQR 1403-3580)	1687 (IQR 970-2162)	200	-275 to1943	0.2
	Serum troponin. NR <34 ng/L	63 (IQR 28-146)			152 (IQR 63-249)	36 (IQR 22-64) **	116	28 to 188	0.006
	Serum ferntm. NR 20-300 ug/L	825 (IQR 417-1403)			1612 (IQR 740-2018)	539 (IQR 340-857) **	1073	93 to 1546	0.01
	White cell count. NR 4-11 x10^^^9/L	5.6 (IQR 3.7-6.4)			4.9 (IQR 3.4-6.2)	5.8 (IQR 4.3-7.0)	0.9	-2.3to1.1	0.3
	Lymphocyte count. NR 1-4 x10^^^9/L	0.7 (IQR 0.5-1.0)			0.5 (IQR 0.4-0.9)	1(IQR 0.6-1.2)	-0.5	-0.5 to 0	0.06
Peak level of COVID-19 clinical biomarker	C-reactive protein. NR<5mgiL	129 (IQR 43-177)			193 (IQR 143-242)	43 (IQR 27-103) **	150	92 to 189	<0.0001
	D-dimer. NR <500 ng/ml	2141(IQR 1479-3640)			3254 (IQR 1894-5540)	1958 (IQR 1347-2951) **	1296	39 to 2849	0.049
	Serum troponin. NR <34 ng/L	84 (IQR 33-175)			181(IQR 105-656)	47 (IQR 22-68) **	134	57 to 523	0.0002
	Serum ferritin. NR 20-300 ug/L	992 (IQR 641-2310)			2332 (IQR 1294-3346)	690 (IQR 573-937) **	1627	475 to 2372	0.001
	White cell count. NR 4-11 x10^^^9/L	7.4 (IQR 5.8-9)			8.6 (IQR 7.4-10.6)	6.9 (IQR 5.8-7.7) **	1.7	0.3 to 4.3	0.03
	Lymphocyte count, nadir. NR 1-4 x10^9/L	0.6 (IQR 0.4-0.9)			0.4 (IQR 0.3-0.6)	0.8 (IQ R0.6-l.0)**	-0.4	-0.7to -0.2	0.0003

Data are numbers (%), median (range) or median (inter-quartile range (IQR)). *mark statistically significant differences between COVID-19 and dialysis control or healthy control cohorts. **mark statistically significant differences between patients with severe and non-severe peak COVID-19 clinical severity. Differences calculated with the Mann-Whitney U test for continuous and Fisher Exact tests for categorical data.

All patients demonstrated typical clinical features of COVID-19 (25–27) (**Table 1** and **Supplementary Figure 1**). Sixteen of the 33 COVID-19 patient cohort had severe disease (**Figure 1A** and **Table 1**). Four patients (12%) died from COVID-19. Levels of clinical biomarkers associated with COVID-19 were higher in the severe compared to non-severe disease cohorts (**Table 1**).

To determine if kidney impairment altered lectin pathway plasma protein concentrations we first compared healthy and dialysis controls. Plasma levels of MAp19 were higher and CL-K1 were lower in dialysis controls (p=0.01), which may be expected given CL-K1 expression in kidney tissue (28) (**Figure 1B** and **Supplementary Table 2**). We next questioned whether lectin pathway protein levels associated with COVID-19 severity at the time of sampling. This approach allowed us to utilise all samples and avoided sample selection bias. However, our data set included repeated samples and different sample numbers in each cohort. We therefore analysed these data with a mixed REML model, the results of which can be interpreted like repeated measures ANOVA. Compared to the dialysis control cohort, samples from patients with non-severe COVID-19 had higher levels of the lectin PRMs M-ficolin (p=0.02) and CL-K1 (p=0.007) and the C3 activation marker C3dg (p=0.02), but lower CL-L1 (p=0.02), MASP-3 (p=0.008) and MAp19 (p=0.008) levels (**Figure 1B** and **Supplementary Table 2**). Compared to non-severe COVID-19, samples from patients with severe COVID-19 had higher H-ficolin (p=0.02), MASP-2 (p=0.03), and MAp19 (p=0.03) levels. MASP-3 levels were lower but this difference did not reach statistical significance after adjustment for multiple comparisons (p=0.07) (**Figure 1B** and **Supplementary Table 2**). Consistent with published data, CRP (p=0.002) and D-dimer (p=0.04) levels were significantly higher in severe disease (**Figure 1C**). These data are summarised in **Figure 1D**.

**Figure 2:**
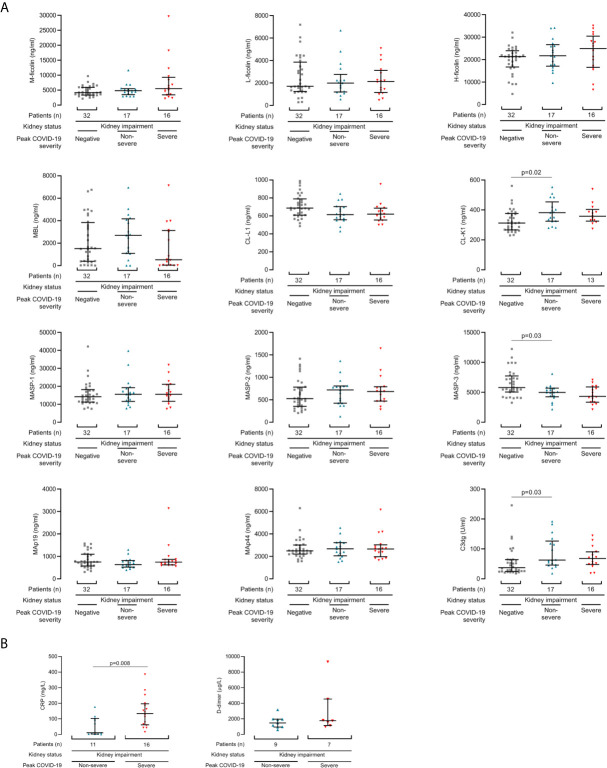
Lectin pathway protein concentrations associate with COVID-19 from the first samples after diagnosis. **(A)** Lectin protein levels from first sample collected after COVID-19 diagnosis in 33 kidney disease patients, of whom 16 developed severe COVID-19 (red triangles) and 17 had non-severe disease (blue triangles). Controls are 32 haemodialysis patients without COVID-19 (grey squares). **(B)** CRP and d-dimer levels from first sample collected after COVID-19 diagnosis in patients with kidney disease and COVID-19. Lines and whiskers show the median and interquartile values. Differences between cohorts were calculated with a Kruskall-Wallis test and follow-up comparison of the mean rank of every column. P values were adjusted for multiple comparisons as described in the methods.

To demonstrate whether lectin protein levels might predict future disease severity, we next examined protein levels from the first samples collected after COVID-19 diagnosis. This allowed comparison of single samples per patient. Samples were collected at median 4 days (IQR 2 to 9 days) from positive SARS-CoV2 swab and diagnosis. From the first sample post diagnosis, plasma CL-K1 (p=0.02) and C3dg (p=0.03) were higher and plasma MASP-3 concentrations were lower (p=0.03) in patients with COVID-19 than dialysis controls (**Figure 2**). We did not detect associations between first sample lectin protein concentrations and COVID-19 severity (**Figure 2**). We also did not detect significant differences from the first collected sample in the 4 patients who died from COVID-19 (**Supplementary Figure 2**).

The COVID-19 population included 6 patients who, despite having significant kidney disease, did not require haemodialysis at enrolment. In contrast, all dialysis control patients were established on chronic intermittent haemodialysis. To establish whether this difference influenced the results, we repeated analyses after exclusion of non-haemodialysis patients. The demographic and clinical characteristics of the haemodialysis COVID-19 cohort were similar to the complete COVID-19 cohort (**Supplementary Table 3**). Also, analyses of the haemodialysis only COVID-19 cohort revealed very similar associations between lectin protein concentrations and COVID-19 (**Supplementary Figures 3–5**).

**Figure 3:**
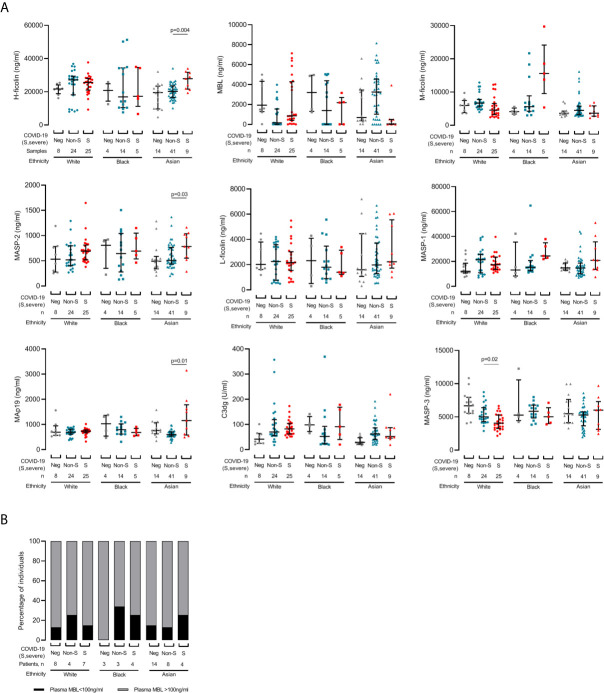
Changes in lectin pathway protein levels during COVID-19 are influenced by ethnicity. **(A)** Samples are grouped by patient self-reported White (circles), Black (squares) or Asian (triangles) ethnicity. 57 samples were collected from 19 White patients, 23 samples were collected from 10 Black patients, and 61 samples were collected from 26 Asian patients. COVID-19 status at sampling was negative (‘Neg’, grey), non-severe (‘Non-S’, blue) or severe (‘S’, red) at sampling. We did not detect differences for CL-L1, CL-K1 and MAp44 (data not shown). Lines and whiskers show the cohort median and interquartile values. Differences between cohorts were calculated with a mixed model for repeated measures and P values adjusted for multiple comparisons as described in the methods. **(B)** Prevalence of MBL deficiency, defined as plasma MBL level less than 100ng/ml, in each ethnicity and severity groups.

Since lectin pathway gene polymorphisms associate with ethnicity and influence protein concentrations, most notably for MBL (13), we next assessed protein levels in all available samples according to self-reported White, Black and Asian ethnicity sub-groups (**Figure 3**). Lectin protein concentrations did not differ between dialysis control cohorts of White, Black or Asian ethnicity (**Figure 3A**) and the proportions of patients with MBL deficiency were similar regardless of either ethnicity or COVID-19 severity (**Figure 3B**). However, amongst individuals of Asian ethnicity, levels of H-ficolin (p=0.004), MASP-2 (p=0.03) and MAp19 (p=0.01) were higher in severe disease (**Figure 3A**). Amongst individuals of White ethnicity, MASP-3 levels were lower in severe compared with non-severe COVID-19 (p=0.02. **Figure 3A**). Taken together, these data showed that associations between lectin pathway proteins and severe and non-severe COVID-19 differed between ethnic groups.

**Figure 4:**
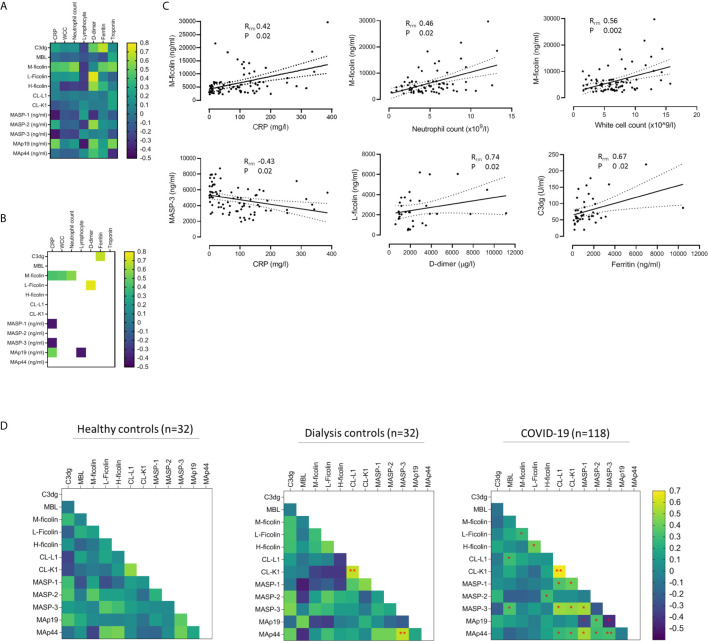
Associations between lectin protein levels and biomarkers of COVID-19 severity. **(A)** Heat map of correlations between lectin proteins and clinical biomarkers in 33 patients with COVID-19. Correlations calculated from 80 samples pairs for CRP, 79 for white cell count (WCC), 75 for neutrophil count and lymphocyte count, 33 for D-dimer, and 40 for troponin and ferritin. **(B)** Heat map of correlations that reached statistical significance after adjusting p-values for multiple analyses. Of these, six correlations had best fit line gradients that were significantly non-zero **(C)**. Solid and dotted lines show the lines of best fit and 95% confidence intervals (95% CI). **(D)** Heat map of correlations between lectin protein levels in 32 healthy controls, 32 haemodialysis patients without COVID-19 (dialysis controls) and 118 samples from 33 patients with kidney impairment and COVID-19. Red stars mark correlations that reached statistical significance after adjusting for multiple analyses. Double red stars mark statistically significant correlations detected in more than one cohort. For the healthy controls and dialysis controls cohorts, we calculated Pearson correlations (R) on log-transformed data. For the COVID-19 cohort, we applied linear mixed models and repeated measures correlation technique (rmcorr) of log-transformed data to calculate correlations (R_rm_) (23).

We next correlated lectin proteins with biomarkers of COVID-19 severity and identified eight correlations that were statistically significant after adjustment for multiple comparisons (**Figures 4A, B**). Of these, the correlation regression slopes were significantly non-zero for six correlations: M-ficolin with CRP, neutrophil count, and white cell count, L-ficolin with D-dimer, MASP-3 with CRP, and C3dg with ferritin (**Figure 4C**). Since lectin proteins circulate as complexes of PRMs with proteases and non-enzymatic proteins, we also performed correlations between lectin components (**Figure 4D** and **Supplementary Figure 6**). CL-L1 and CL-K1 levels correlated which was expected since these proteins circulate as polypeptide hetero-trimers (29) (**Figure 4D** and **Supplementary Figure 6A**). For most protein pairs, correlations were similar across all cohorts (**Supplementary Figure 6**). Notably, correlations between MASP-1 and Map19 and between MASP-3 and MAp19 were negative in COVID-19 patients but positive in dialysis controls (**Figure 4D** and **Supplementary Figure 6B**) providing further evidence that lectin protein levels are altered during COVID-19 infection. We repeated the analysis in only samples taken during severe COVID-19 and demonstrated similar correlations to the total COVID-19 cohort (**Supplementary Figure 7**). This implies that correlations between lectin pathway proteins are influenced more by the presence of COVID-19 infection than COVID-19 severity.

## Discussion

We questioned whether lectin complement pathway protein levels associated with COVID-19 severity and whether this was influenced by ethnicity in patients with kidney disease. We demonstrated associations between COVID-19 and circulating levels of lectin PRMs, (M- and H-ficolin, CL-L1 and CL-K1) and proteases (MASP-2, MAp19 and MASP-3). We detected increased plasma H-ficolin, MASP-2 and MAp19 in severe compared to non-severe COVID-19. Also, increased CL-K1, C3dg and CRP, and reduced MASP-3 were detectable from first available samples, indicating associations between lectin complement pathway activation early in COVID-19 disease course. Together, these data indicate lectin pathway protein concentration and activity contributes to COVID-19 pathogenesis. We also demonstrated differences in lectin complement protein levels between non-infected haemodialysis patients and healthy controls, which suggests kidney failure and haemodialysis also influence lectin pathway activity and warrants further investigation.

Ethnicity analyses revealed different lectin protein patterns and associations with COVID-19 severity. Increased levels of the PRM H-ficolin and associated proteases and proteins MASP-2 and MAp19 associated with severe disease in Asian patients, and the association of reduced MASP-3 with severe COVID-19 was detected in White patients only. Additionally, we noticed differences that did not reach statistical significance after multiple comparison; we detected lower MBL levels in severe than non-severe COVID-19 in Asian individuals (adjusted p-value = 0.08); and amongst individuals of Black ethnicity, levels of M-ficolin and MASP-1 were higher in severe disease (**Figure 3A**, adjusted p-values 0.06 for M-ficolin and 0.08 for MASP-1). Although these results were derived from small numbers of patients and should be interpreted with caution, they suggest the lectin pathway proteins that contribute to COVID-19 pathogenesis are influenced by ethnicity. This is important evidence that immunological variants, that can be quantified and potentially targeted therapeutically, contribute to the additional risk of severe COVID-19 in non-White ethnicity groups (1, 2, 30).

Evidence of specific lectin pathway activation is very difficult to identify in clinical samples from infected patients because products of lectin pathway activity, such as C4a and C4d, can also result from classical pathway activation. Therefore, as a surrogate of complement pathway activity, we demonstrated associations between circulating C3dg, a marker of down-stream complement activation (the assay detects both free and protein-bound C3dg), and COVID-19. We also showed that M-ficolin, L-ficolin, MASP-3 and C3dg correlated with clinical biomarkers of inflammation and thrombosis known to associate with COVID-19 infection and severity (**Figure 4**). Finally, we demonstrated that relationships between lectin proteins are altered in COVID-19 (**Figure 4** and **Supplementary Figure 6**). Together these data indicate that circulating concentrations of lectin pathway proteins contribute to complement activity, inflammation and severity of COVID-19. In addition to providing pathogenic insight, our research could direct the re-purposing of existing therapeutic agents that target lectin pathway activity, such as the MASP-2 inhibitor Narsoplimab, to clinical trials of COVID-19.

Our study provides the first comprehensive analysis of lectin pathway activity in COVID-19. We conceived, designed and collected samples for our research during the worsening first peak of the COVID-19 pandemic. This provided valuable serial samples from individuals with multiple risk factors for severe COVID-19, many of whom were enrolled with mild symptoms early in disease course when screened at dialysis units. However, due to limited resources sample collection and timing was non-uniform. We also included patients with a range of demographic and clinical characteristics, including individuals with significant kidney impairment but not established on chronic haemodialysis. Consequently, we adopted statistical tools to adjust for repeated and missing samples and repeated analyses after excluding non-haemodialysis patients. Our analysis is also limited by the unavailability of a plasma marker of specific lectin complement pathway activation.

We do not know the causes of lectin protein concentration differences in COVID-19, but speculate this is secondary to lectin pathway activation within the lung and other infected tissues. Experimental models suggest MBL can bind the SARS-CoV spike protein (18), which is densely decorated with heterogeneous N-linked glycans (31). The majority of the SARS-CoV glycosylation sequences are conserved in SARS-CoV-2 (32). However, the interaction of lectin pathway PRMs with SARS-CoV-2 has not been demonstrated. Additionally, lectin pathway activation could occur *via* mechanisms not specific to SARS-CoV-2, such as binding to glycan structures on injured cells and inflamed tissue. Consistent with this and in line with our results, increased plasma H-ficolin, MASP-2 and MAp19 concentrations have been documented in severely ill patients with septic shock (15). However, whether severe inflammation drives increased H-ficolin, MASP-2 and MAp19 levels or pre-existing protein concentrations predispose individuals to severe sepsis is unclear. Also similar to our data, lower plasma MASP-3 concentrations have been identified in patients with bacterial sepsis (15). MASP-3 is the exclusive physiological activator of pro-factor D to factor D, which is a key enzyme in alternative complement pathway activation (33). Therefore, lung deposition of MASP-3 with subsequent alternative pathway activation and inflammation could explain associations between reduced circulating MASP-3, lung inflammation and COVID-19 severity. However, tissue-level complement analysis will be required to determine the contribution of complement to lung injury. Interestingly, multiple studies have demonstrated associations between COVID-19 and increased circulating concentrations of the anaphylatoxins C3a and C5a, C5a receptor expression, and the terminal pathway effector molecule C5b9 (7–10, 34, 35). Therefore, down-stream complement activity, which could be triggered by the lectin and alternative pathways, is very likely to be important to COVID-19 pathogenesis.

In summary, analysis of the lectin complement pathway in CKD patients with COVID-19 has highlighted multiple associations, some of which are unique to individuals of Asian ethnicity. These data require further research to identify the mechanisms of lectin pathway activation and to validate lectin pathway inhibition as a potential treatment for severe COVID-19.

## Data Availability Statement

The raw data supporting the conclusions of this article will be made available by the authors, without undue reservation.

## Ethics Statement

The studies involving human participants were reviewed and approved by Health Research Authority, Research Ethics Committee (reference: 20/WA/0123). The patients/participants provided their written informed consent to participate in this study.

## Author Contributions

NM-T conceived and designed the research, acquired samples and data, analyzed data and wrote the manuscript. AT, AH and ST conducted lectin protein quantification experiments, wrote and reviewed the manuscript. MCP acquired samples and wrote and reviewed the manuscript. JG and JP assisted with statistical analysis and wrote the manuscript. CC, MP, SM, ES, and LL collected and processed samples. DCT, MW and MB collected samples and data and contributed to the manuscript. All authors contributed to the article and approved the submitted version.

## Funding

This research was partly funded by Community Jameel and the Imperial President’s Excellence Fund and by a UKRI-DHSC COVID-19 Rapid Response Rolling Call (MR/V027638/1). We also acknowledge a contribution from UKRI/NIHR through the UK Coronavirus Immunology Consortium (UK-CIC) and the National Institute for Health Research (NIHR) Biomedical Research Centre (BRC) based at Imperial College Healthcare NHS Trust and Imperial College London. JP is supported by UKRI Innovation Fellowship at Health Data Research UK (MR/S004068/2). DCT is funded by a Stage 2 Wellcome-Beit Prize Clinical Research Career Development Fellowship (20661206617/A/17/Z and 206617/A/17/A) and the Sidharth Burman endowment. MP is a Wellcome Trust Senior Fellow in Clinical Science (212252/Z/18/Z). NM-T and ES are supported by Wellcome Trust and Imperial College London Research Fellowships, and CC by an Auchi Clinical Research Fellowship.

## Disclaimer

The views expressed are those of the author(s) and not necessarily those of the NHS, the NIHR or the Department of Health.

## Conflict of Interest

The authors declare that the research was conducted in the absence of any commercial or financial relationships that could be construed as a potential conflict of interest.

## References

[1] Williamson EJ Walker AJ Bhaskaran K Bacon S Bates C Morton CE. Factors Associated With COVID-19-related Death Using Opensafely. Nature (2020) 584(7821):430–6. 10.1038/s41586-020-2521-4 PMC7611074 32640463

[2] Raisi-Estabragh Z McCracken C Bethell MS Cooper J Cooper C Caulfield MJ. Greater Risk of Severe COVID-19 in Black, Asian and Minority Ethnic Populations is Not Explained by Cardiometabolic, Socioeconomic or Behavioural Factors, or by 25(OH)-Vitamin D Status: Study of 1326 Cases From the UK Biobank. J Public Health (Oxf) (2020) 42(3):451–60. 10.1093/pubmed/fdaa095 PMC7449237 32556213

[3] Cheng Y Luo R Wang K Zhang M Wang Z Dong L. Kidney Disease is Associated With in-Hospital Death of Patients With COVID-19. Kidney Int (2020) 97(5):829–38. 10.1016/j.kint.2020.03.005 PMC7110296 32247631

[4] Ali YM Lynch NJ Haleem KS Fujita T Endo Y Hansen S. The Lectin Pathway of Complement Activation is a Critical Component of the Innate Immune Response to Pneumococcal Infection. PLoS Pathog (2012) 8(7):e1002793. 10.1371/journal.ppat.1002793 22792067 PMC3390405

[5] Ekdahl KN Teramura Y Hamad OA Asif S Duehrkop C Fromell K. Dangerous Liaisons: Complement, Coagulation, and Kallikrein/Kinin Cross-Talk Act as a Linchpin in the Events Leading to Thromboinflammation. Immunol Rev (2016) 274(1):245–69. 10.1111/imr.12471 27782319

[6] Conway EM Pryzdial ELG. Is the COVID-19 Thrombotic Catastrophe Complement-Connected? J Thromb Haemost (2020) 18:2812–22. 10.1111/jth.15050 PMC7436532 32762081

[7] Cugno M Meroni PL Gualtierotti R Griffini S Grovetti E Torri A. Complement Activation in Patients With COVID-19: A Novel Therapeutic Target. J Allergy Clin Immunol (2020) 146(1):215–7. 10.1016/j.jaci.2020.05.006 PMC7224678 32417135

[8] Holter JC Pischke SE de Boer E Lind A Jenum S Holten AR. Systemic Complement Activation is Associated With Respiratory Failure in COVID-19 Hospitalized Patients. Proc Natl Acad Sci U S A (2020) 117(40):25018–25. 10.1073/pnas.2010540117 PMC7547220 32943538

[9] Carvelli J Demaria O Vely F Batista L Chouaki Benmansour N Fares J. Association of COVID-19 Inflammation With Activation of the C5a-C5aR1 Axis. Nature (2020) 588:146–50. 10.1038/s41586-020-2600-6 PMC7116884 32726800

[10] Prendecki M Clarke C Medjeral-Thomas N McAdoo SP Sandhu E Peters JE. Temporal Changes in Complement Activation in Haemodialysis Patients With COVID-19 as a Predictor of Disease Progression. Clin Kidney J (2020) 13(5):889–96. 10.1093/ckj/sfaa192 PMC7577776 33123364

[11] Degn SE Thiel S Jensenius JC. New Perspectives on Mannan-Binding Lectin-Mediated Complement Activation. Immunobiology (2007) 212(4-5):301–11. 10.1016/j.imbio.2006.12.004 17544815

[12] Garred P Genster N Pilely K Bayarri-Olmos R Rosbjerg A Ma YJ. A Journey Through the Lectin Pathway of complement-MBL and Beyond. Immunol Rev (2016) 274(1):74–97. 10.1111/imr.12468 27782323

[13] Garred P Larsen F Seyfarth J Fujita R Madsen HO. Mannose-Binding Lectin and its Genetic Variants. Genes Immun (2006) 7(2):85–94. 10.1038/sj.gene.6364283 16395391

[14] Garred P Thiel S Madsen HO Ryder LP Jensenius JC Svejgaard A. Gene Frequency and Partial Protein Characterization of an Allelic Variant of Mannan Binding Protein Associated With Low Serum Concentrations. Clin Exp Immunol (1992) 90(3):517–21. 10.1111/j.1365-2249.1992.tb05876.x PMC1554582 1458688

[15] Larsen JB Laursen MA Hvas CL Larsen KM Thiel S Hvas AM. Reduced Mannose-Binding Lectin-Associated Serine Protease (MASP)-1 is Associated With Disturbed Coagulation in Septic Shock. Thromb Haemost (2019) 119(6):952–61. 10.1055/s-0039-1685140 30986866

[16] Gao DN Zhang Y Ren YB Kang J Jiang L Feng Z. Relationship of Serum Mannose-Binding Lectin Levels With the Development of Sepsis: A Meta-Analysis. Inflammation (2015) 38(1):338–47. 10.1007/s10753-014-0037-5 25323207

[17] LaFon DC Thiel S Kim YI Dransfield MT Nahm MH. Classical and Lectin Complement Pathways and Markers of Inflammation for Investigation of Susceptibility to Infections Among Healthy Older Adults. Immun Ageing (2020) 17:18. 10.1186/s12979-020-00189-7 32536956 PMC7285792

[18] Zhou Y Lu K Pfefferle S Bertram S Glowacka I Drosten C. A Single Asparagine-Linked Glycosylation Site of the Severe Acute Respiratory Syndrome Coronavirus Spike Glycoprotein Facilitates Inhibition by Mannose-Binding Lectin Through Multiple Mechanisms. J Virol (2010) 84(17):8753–64. 10.1128/JVI.00554-10 PMC2919028 20573835

[19] Eriksson O Hultstrom M Persson B Lipcsey M Ekdahl KN Nilsson B. Mannose-Binding Lectin is Associated With Thrombosis and Coagulopathy in Critically Ill COVID-19 Patients. Thromb Haemost (2020) 120(12):1720–4. 10.1055/s-0040-1715835 PMC7869044 32871607

[20] Wu Z McGoogan JM. Characteristics of and Important Lessons From the Coronavirus Disease 2019 (Covid-19) Outbreak in China: Summary of a Report of 72314 Cases From the Chinese Center for Disease Control and Prevention. JAMA (2020) 323(13):1239–42. 10.1001/jama.2020.2648 32091533

[21] Troldborg A Hansen A Hansen SW Jensenius JC Stengaard-Pedersen K Thiel S. Lectin Complement Pathway Proteins in Healthy Individuals. Clin Exp Immunol (2017) 188(1):138–47. 10.1111/cei.12909 PMC5343365 27925159

[22] Troldborg A Halkjaer L Pedersen H Hansen A Loft AG Lindegaard H. Complement Activation in Human Autoimmune Diseases and Mouse Models; Employing a Sandwich Immunoassay Specific for C3dg. J Immunol Methods (2020) 486:112866. 10.1016/j.jim.2020.112866 32941885

[23] Bakdash JZ Marusich LR. Repeated Measures Correlation. Front Psychol (2017) 8:456. 10.3389/fpsyg.2017.00456 28439244 PMC5383908

[24] Benjamini Y KA Yekutieli D. Adaptive Linear Step-Up Procedures That Control the False Discovery Rate. Biometrika (2006) 96:491–507. 10.1093/biomet/93.3.491

[25] Zhou F Yu T Du R Fan G Liu Y Liu Z. Clinical Course and Risk Factors for Mortality of Adult Inpatients With COVID-19 in Wuhan, China: A Retrospective Cohort Study. Lancet (2020) 395(10229):1054–62. 10.1016/S0140-6736(20)30566-3 PMC7270627 32171076

[26] Goyal P Choi JJ Pinheiro LC Schenck EJ Chen R Jabri A. Clinical Characteristics of Covid-19 in New York City. N Engl J Med (2020) 382(24):2372–4. 10.1056/NEJMc2010419 PMC7182018 32302078

[27] Guan WJ Ni ZY Hu Y Liang WH Ou CQ He JX. Clinical Characteristics of Coronavirus Disease 2019 in China. N Engl J Med (2020) 382(18):1708–20. 10.1056/NEJMoa2002032 PMC7092819 32109013

[28] Keshi H Sakamoto T Kawai T Ohtani K Katoh T Jang SJ. Identification and Characterization of a Novel Human Collectin CL-K1. Microbiol Immunol (2006) 50(12):1001–13. 10.1111/j.1348-0421.2006.tb03868.x 17179669

[29] Henriksen ML Brandt J Andrieu JP Nielsen C Jensen PH Holmskov U. Heteromeric Complexes of Native Collectin Kidney 1 and Collectin Liver 1 are Found in the Circulation With MASPs and Activate the Complement System. J Immunol (2013) 191(12):6117–27. 10.4049/jimmunol.1302121 24174618

[30] Kirby T. Evidence Mounts on the Disproportionate Effect of COVID-19 on Ethnic Minorities. Lancet Respir Med (2020) 8(6):547–8. 10.1016/S2213-2600(20)30228-9 PMC7211498 32401711

[31] Walls AC Tortorici MA Frenz B Snijder J Li W Rey FA. Glycan Shield and Epitope Masking of a Coronavirus Spike Protein Observed by Cryo-Electron Microscopy. Nat Struct Mol Biol (2016) 23(10):899–905. 10.1038/nsmb.3293 27617430 PMC5515730

[32] Walls AC Park YJ Tortorici MA Wall A McGuire AT Veesler D. Structure, Function, and Antigenicity of the SARS-CoV-2 Spike Glycoprotein. Cell (2020) 183(6):1735. 10.1016/j.cell.2020.11.032 33306958 PMC7833104

[33] Dobo J Szakacs D Oroszlan G Kortvely E Kiss B Boros E. MASP-3 is the Exclusive Pro-Factor D Activator in Resting Blood: The Lectin and the Alternative Complement Pathways are Fundamentally Linked. Sci Rep (2016) 6:31877. 10.1038/srep31877 27535802 PMC4989169

[34] Ramlall V Thangaraj PM Meydan C Foox J Butler D Kim J. Immune Complement and Coagulation Dysfunction in Adverse Outcomes of SARS-CoV-2 Infection. Nat Med (2020) 26(10):1609–15. 10.1038/s41591-020-1021-2 PMC7809634 32747830

[35] Messner CB Demichev V Wendisch D Michalick L White M Freiwald A. Ultra-High-Throughput Clinical Proteomics Reveals Classifiers of COVID-19 Infection. Cell Syst (2020) 11(1):11–24.e4. 10.1016/j.cels.2020.05.012 32619549 PMC7264033

