# Supplementary material for: Plasma Lectin Pathway Complement Proteins in Patients With COVID-19 and Renal Disease
Source: Front Immunol. 2021 Apr 29;12:671052. doi: 10.3389/fimmu.2021.671052 (PMC8118695; doi:10.3389/fimmu.2021.671052)
Supplement: Supplementary file 1 [file Presentation_1.pdf]

Lectin complement activation signatures associate with COVID-19 severity

Supplementary data

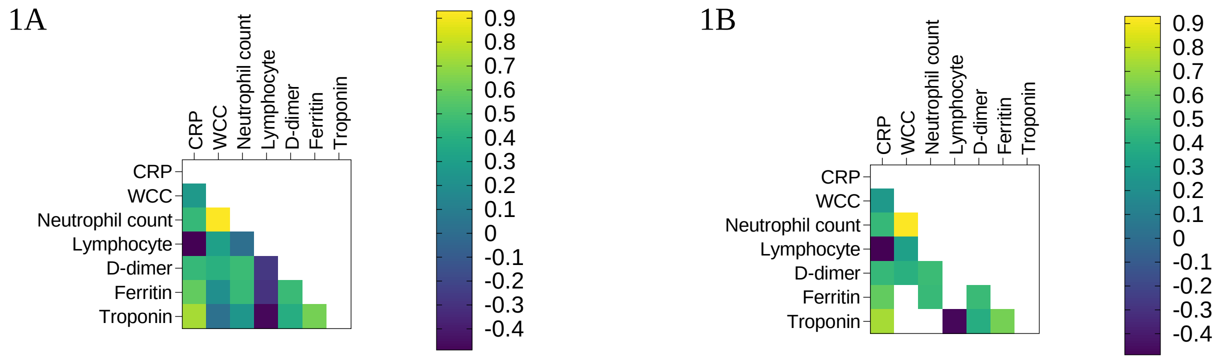

Supplementary Figure 1: Clinical markers of COVID-19 severity in all samples from patients with COVID-19.

A. R-values calculated using Spearman rank correlation of 81 samples form 33 patients.

B. Correlations that did not reach statistical significance after adjustment with the method of Benjamini and Hochberg and false discovery rate (Q) of 5% have been removed.

		COVID-19	Severe COVID-19	Non-severe COVID-19
Number of patients		33	16 (Severe = 9, critical = 7)	17 (Mild = 11, moderate = 6)
Days symptom to first sample		5 (4-9)	6 (4-10)	5 (2-9)
Days diagnostic swab to first sample		2 (2-4)	3 (1-4)	2 (2-5)
Total samples		118	71	47
Number of samples per patient	7	1	1	0
	6	6	6	0
	5	1	1	0
	4	4	4	0
	3	13	0	13
	2	7	3	4
	1	1	1	0
Days first symptom to samples		14 (9-21)	16 (11-25)	12 (8-16)

Supplementary Table 1: Serial sample collection from patients with COVID-19.
Data are median (interquartile range).

Cohorts and proteins	Healthy control (A)	Dialysis control (B)	Non-severe COVID-19 (C)	Severe COVID-19 (D)	Comparison	Difference of medians	95% CI	P –value, adjusted
Individuals	32	32	17	16				
Samples (n)	32	32	79	39				
MBL, ng/ml	950 (312-2488)	1519 (388-3827)	1874 (21-4027)	605 (55-3452)				
M-ficolin, ng/ml	3671 (3027-4664)	4199 (3381-5984)	5440 (3925-6885)	4905 (3248-6902)	B and C	1241	84 to 1794	0.02
L-ficolin, ng/ml	2708 (2338-3205)	1724 (1253-3868)	2049 (1217-3620)	2167 (1453-3094)				
H-ficolin, ng/ml	18829 (13711-22066)	21374 (16791-24001)	21467 (15370-51268)	25587 (20272-28989)	C and D	4120	408 to 6411	0.02
					B and D	4213	1933 to 7466	0.003
CL-L1, ng/ml	672 (607-703)	686 (608-789)	624 (534-734)	617 (569-688)	B and C	-62	-125 to -19	0.02
CL-K1, ng/ml	375 (336-428)	313 (267-377)	369 (321-437)	360 (318-393)	A and B	-62	-90 to 029	0.01
					B and C	56	23 to 87	0.007
MASP-1, ng/ml	12830 (11414-13748)	14331 (11302-18268)	15494 (11601-21835)	17919 (13814-24317)				
MASP-2, ng/ml	459 (320-774)	529 (356-785)	521 (402-799)	698 (523-852)	C and D	177	38 to 239	0.03
MASP-3, ng/ml	7292 (6040-8424)	5813 (5071-7753)	5308 (4246-5330)	4127 (3365-5868)	B and C	-505	-1803 to -176	0.008
					C and D	-1181	-1423 to -124	0.07
MAp19, ng/ml	445 (373-506)	756 (576-1101)	666 (566-774)	735 (621-833)	A and B	311	212 to 422	<0.0001
					B and C	-90	-216 to -9	0.008
					C and D	69	7 to 143	0.03
MAp44, ng/ml	2512 (2128-2789)	2503 (2220-3024)	2564 (2107-2982)	2745 (2175-3483)				
C3dg, U/ml	30 (24-37)	38 (25-65)	63 (41-99)	79 (57-103)	B and C	25	5 to 35	0.02
CRP , mg/L	NA	NA	31 (8-103) n = 43	114 (55-174) n = 37	C and D	83	33 to 105	0.002
D-dimer, µg/L	NA	NA	1772 (994-1997) n = 17	3187 (1655-4378) n = 16	C and D	1415	218 to 2542	0.04

Supplementary Table 2: Lectin complement protein plasma levels in healthy individuals, individuals with kidney failure and kidney failure patients with severe and non-severe COVID-19 at the time of sampling.

Median (interquartile range) values for samples taken from cohorts of healthy individuals without COVID-19 (Health control), patients with kidney failure but no COVID-19 (dialysis controls) at sampling and kidney failure patients with COVID-19, separated by the clinical severity of COVID-19 at sampling (Non-severe COVID-19 and Severe COVID-19).

Differences between cohorts were calculated with a mixed model for repeated measures that uses a compound symmetry covariance matrix and is fit using Restricted Maximum Likelihood (REML). We applied the Geisser-Greenhouse correction to our data for non-sphericity. We adjusted p-values for multiple comparisons using the method of Benjamini and Hochberg with a false discovery rate (Q) of 5%.

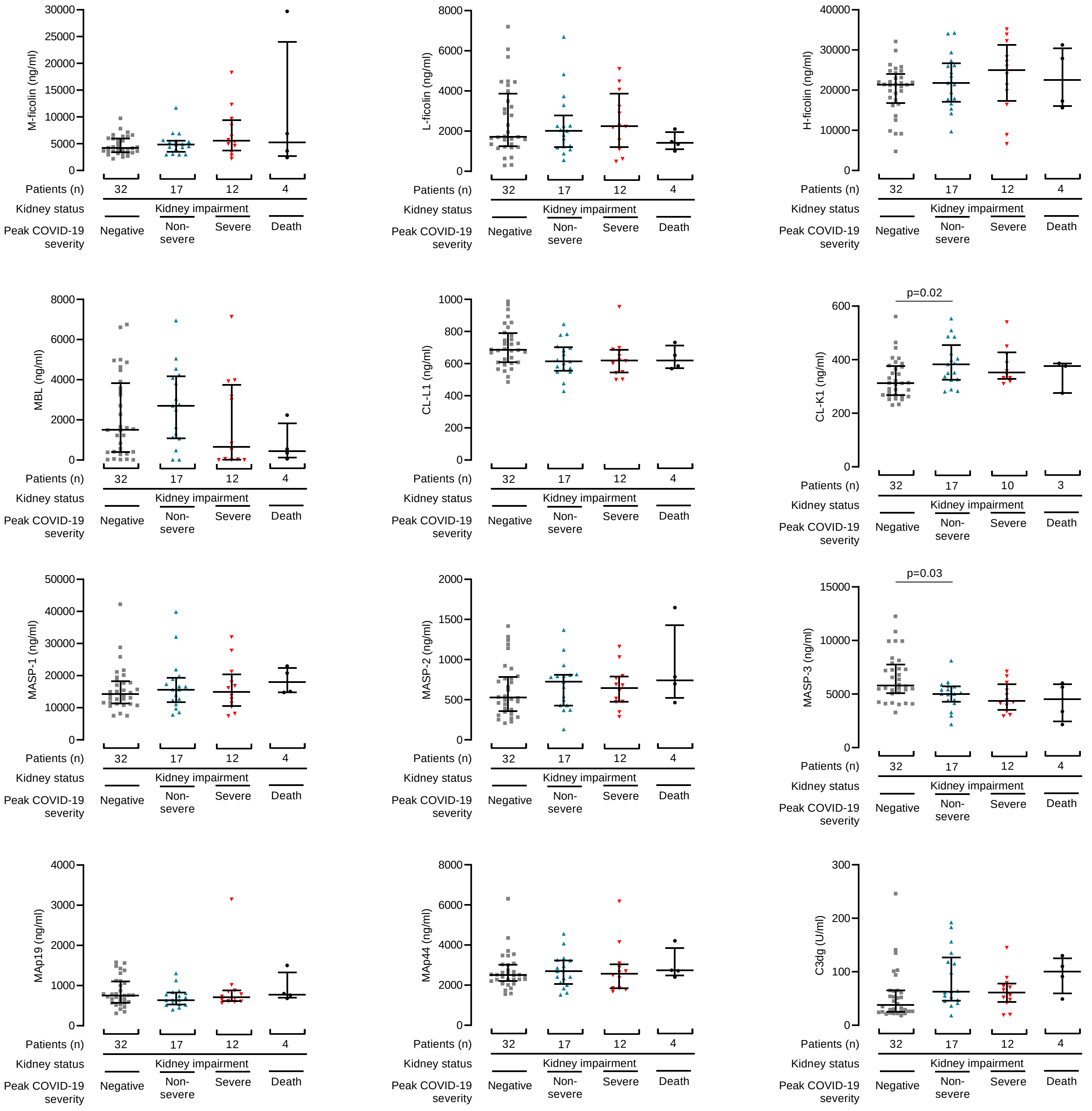

Supplementary Figure 2: Lectin pathway protein concentrations from the first samples after COVID-19 diagnosis and death from COVID-19. Lectin protein levels from first sample collected after COVID-19 diagnosis in 27 haemodialysis patients with COVID-19, of whom 4 diead from COVID-19 (black circles), 12 developed severe COVID-19 (red triangles) and 17 had non-severe disease (blue triangles). Controls are 32 haemodialysis patients without COVID-19 (grey squares). Lines and whiskers show the median and interquartile values. Differences between cohorts were calculated with a Kruskall-Wallis test and follow-up comparison of the mean rank of every column. P values were adjusted for multiple comparisons.

		COVID-19	Dialysis controls	Healthy controls	Severe COVID-19	Non-severe COVID-19	Difference	95% CI	p
	Number	27	32	32	11	16			
	Age, years.	73 (range 40-88)	62 (range 19-86) *				11	4-19	0.004
				48 (range 28-63) *			24	18-30	<0.0001
					66 (44-88)	74 (40-84)			
	Male	17 (63)	19 (59)	17 (53)	7 (64)	10 (63)			
Ethnicity	BAME	18 (67)	24 (75)	20 (63)	6 (54)	12 (75)			
	- Black	6 (22)	3 (9)	6 (19)	3 (27)	3 (18)			
	- Asian	10 (37)	14 (44)	14 (44)	3 (27)	7 (47)			
	- White	9 (33)	8 (25)	12 (37)	5 (46)	4 (24)			
	- Other	2 (7)	7 (22)	0 (0)	0 (6)	2 (12)			
Kidney disease	Diabetic nephropathy	11 (41)	13 (41)		7 (44)	6 (35)			
	Hypertension	2 (7)	0 (0)		1 (6)	2 (12)			
	Glomerulonephritis	4 (14)	8 (25)		1 (6)	3 (18)			
	Genetic	1 (4)	1 (3)		1 (6)	1 (6)			
	Unknown	3 (11)	9 (28)		3 (19)	2 (12)			
	Other	6 (22)	1 (3)		3 (19)	3 (18)			
Co-morbidities	Ischaemic heart disease	14 (52)	15 (47)		7 (44)	10 (59)			
	Current smoking	0 (0)	2 (6)		0 (0)	0(0)			
	Ex-smoker	19 (70)	24 (75)		11 (69)	11 (65)			
	Type 2 diabetes mellitus	12 (44)	15 (47)		8 (50)	7 (41)			
	Antihypertensive medications	22 (81)	23 (72)		13 (81)	15 (88)			
	Current immunosuppression	5 (19)	2 (6)		4 (25)	4 (24)			
	Chronic obstructive pulmonary disease	2 (7)	1 (3)		1 (6)	1 (6)			
COVID-19 progression	Required hospitalisation	11 (41)			16 (100)	1 (6) **			<0.0001
	Died from COVID-19	3 (11)			4 (25)	0 (0) **			0.04
COVID-19 clinical biomarker at diagnostic swab	C-reactive protein. NR<5 mg/L	43 (IQR 16-93)			60 (IQR 24-138)	29 (IQR 6-77)	31	-79 to 11	0.1
	D-dimer. NR <500 ng/ml	1818 (IQR 1087-2475)			1887 (IQR 1700-2973)	1479 (IQR 958-2064)	408	-1567 to 323	0.2
	Serum troponin. NR <34 ng/L	58 (IQR 27-104)			146 (IQR 63-168)	35 (IQR 22-65) **	111	15 to 134	0.01
	Serum ferritin. NR 20-300 ug/L	841 (IQR 445-1531)			1938 (IQR 1241-2294)	520 (IQR 330-878) **	1418	529 to 1772	0.0009
	White cell count. NR 4-11 $x10^9$/L	5.5 (IQR 3.8-6.2)			4.3 (IQR 2.9-6.0)	5.8 (IQR 4.3-6.6)	1.5	-0.6 to 2.7	0.2
	Lymphocyte count. NR 1-4 $x10^9$/L	0.9 (IQR 0.5-1.1)			0.5 (IQR 0.4-0.9)	1 (IQR 0.7-1.3) **	-0.5	-0.7 to -0.1	0.03
Peak level of COVID-19 clinical biomarker	C-reactive protein. NR<5 mg/L	124 (IQR 37-168)			171 (IQR 140-228)	40 (IQR 24-95) **	131	91 to 192	<0.0001
	D-dimer. NR <500 ng/ml	1986 (IQR 1450-3552)			3464 (IQR 1864-4334)	1927 (IQR 1317-3005) **	1537	30 to 2844	0.049
	Serum troponin. NR <34 ng/L	69 (IQR 30-114)			152 (IQR 105-232)	40 (IQR 22-69) **	112	44 to 171	0.0004
	Serum ferritin. NR 20-300 ug/L	992 (IQR 639-2206)			2835 (IQR 1637-3408)	666 (IQR 543-938) **	2169	684 to 2646	0.0006
	White cell count. NR 4-11 $x10^9$/L	7.4 (IQR 5.9-9.4)			9.8 (IQR 7.7-11.1)	6.7 (IQR 5.6-7.5) **	3.1	0.9 to 5.1	0.006
	Lymphocyte count, nadir	0.7 (IQR 0.4-1.0)			0.4 (IQR 0.3-0.6)	0.9 (IQR 0.7-1.1) **	-0.5	-0.7 to -0.2	0.002

Supplementary Table 3: Characteristics of haemodialysis patients with COVID-19 and control cohorts.

Data are numbers (%), median (range) or median (inter-quartile range (IQR)). * mark statistically significant differences between COVID-19 and dialysis control or healthy control cohorts. ** mark statistically significant differences between patients with severe and non-severe peak COVID-19 clinical severity. Differences calculated with the Mann-Whitney U test for continuous and Fisher Exact tests for categorical data.

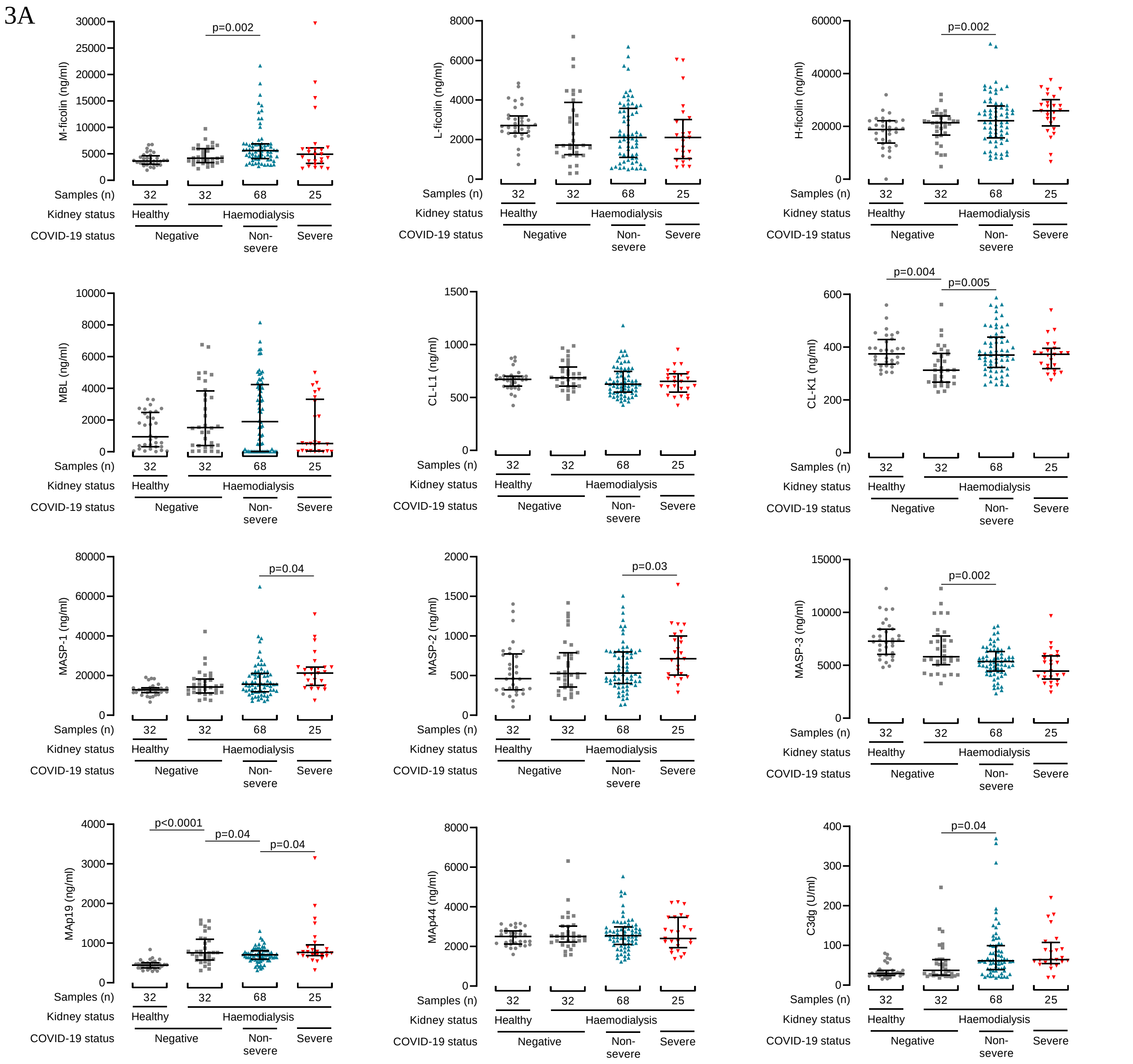

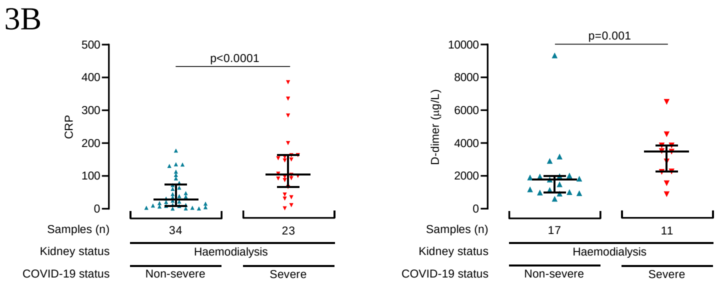

3C

Associations with higher (↑) or lower (↓) concentrations	Pattern recognition molecules	MBL associated serine proteases and proteins	Complement activity
Dialysis control vs healthy control	CL-K1 ↓	MAp19 ↑	
Non-severe COVID-19 vs dialysis control	M-ficolin ↑<br>H-ficolin ↑<br>CL-K1 ↑	MASP-3 ↓<br>MAp-19 ↓	C3dg ↑
Severe COVID-19 vs non-severe COVID-19		MASP-1 ↑<br>MASP-2 ↑<br>MAp19 ↑	

Supplementary Figure 3: Lectin complement protein plasma levels in haemodialysis patients with non-severe and severe COVID-19, haemodialysis patients without COVID-19 and healthy individuals. (A) Lectin protein levels in 93 samples from 27 haemodialysis patients with COVID-19. 25 samples were from patients with severe (red triangles) and 68 samples were from patients with non-severe (blue triangles) COVID-19 at sampling. Controls are 32 dialysis patients without COVID-19 (dialysis control cohort, grey squares) and 32 healthy individuals (healthy control cohort, grey circles). (B) CRP and d-dimer levels in haemodialysis patients with COVID-19. Lines and whiskers show the median and interquartile values. Differences between cohorts were calculated with a mixed model for repeated measures and P values adjusted for multiple comparisons as described in the methods. (C) Summary of significant associations identified.

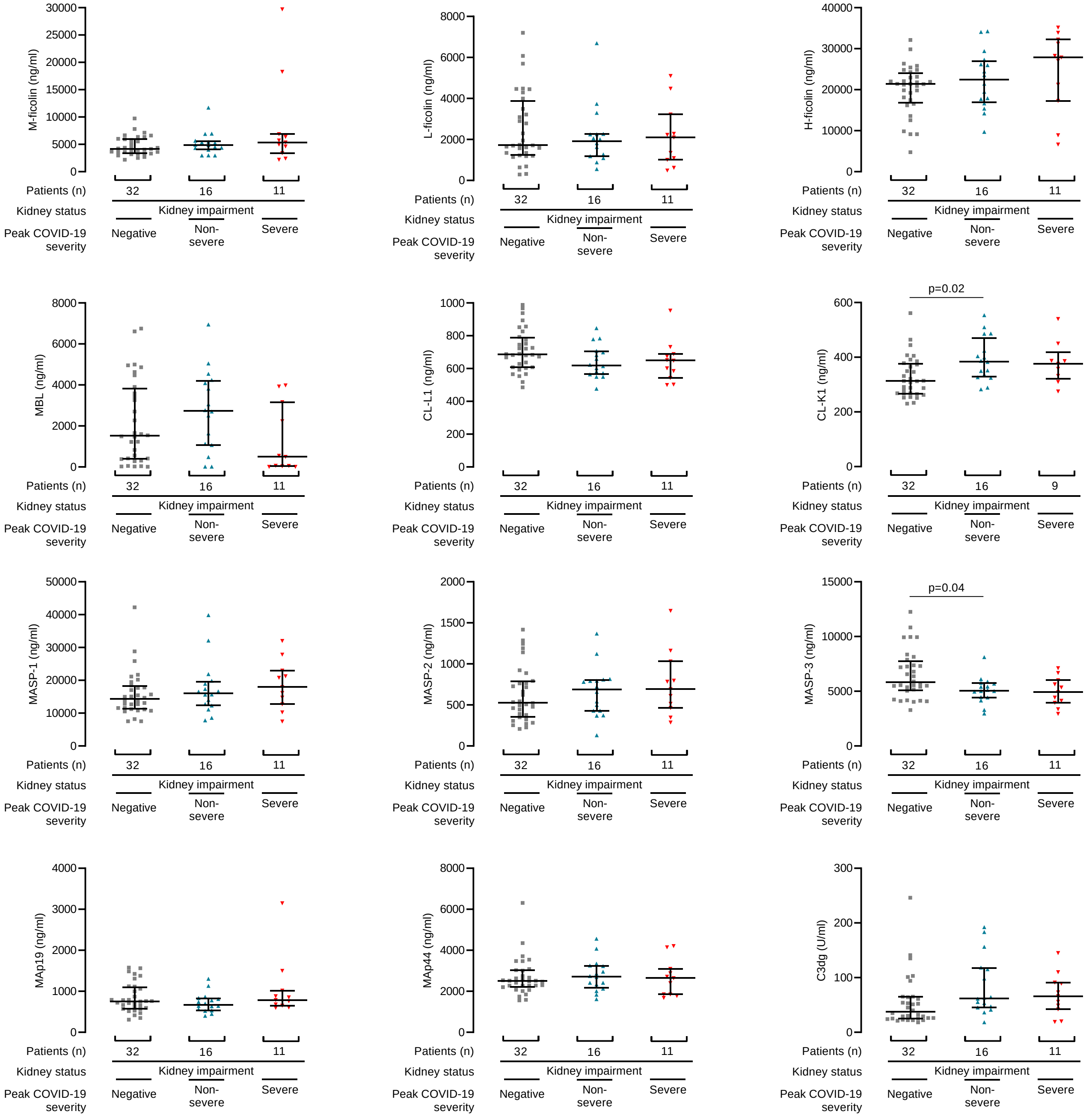

Supplementary Figure 4: Lectin pathway protein concentrations from the first samples after COVID-19 diagnosis associate with COVID-19 in haemodialysis patients. Lectin protein levels from first sample collected after COVID-19 diagnosis in 27 haemodialysis patients with COVID-19, of whom 11 developed severe COVID-19 (red triangles) and 16 had non-severe disease (blue triangles). Controls are 32 haemodialysis patients without COVID-19 (grey squares). Lines and whiskers show the median and interquartile values. Differences between cohorts were calculated with a Kruskall-Wallis test and follow-up comparison of the mean rank of every column. P values were adjusted for multiple comparisons.

Analysis of all available samples

Complete COVID-19 cohort

Associations with higher (↑) or lower (↓) concentrations	Pattern recognition molecules	MBL associated serine proteases and proteins	Complement activity
Dialysis control vs healthy control	CL-K1 ↓	MAp19 ↑	
Non-severe COVID-19 vs dialysis control	M-ficolin ↑<br>CL-K1 ↑<br>CL-L1 ↓	MASP-3 ↓<br>MAp-19 ↓	C3dg ↑
Severe COVID-19 vs non-severe COVID-19	H-ficolin ↑	MASP-2 ↑<br>MAp19 ↑<br>MASP-3 ↓	

COVID-19 haemodialysis patients only

Associations with higher (↑) or lower (↓) concentrations	Pattern recognition molecules	MBL associated serine proteases and proteins	Complement activity
Dialysis control vs healthy control	CL-K1 ↓	MAp19 ↑	
Non-severe COVID-19 vs dialysis control	M-ficolin ↑<br>H-ficolin ↑<br>CL-K1 ↑	MASP-3 ↓<br>MAp-19 ↓	C3dg ↑
Severe COVID-19 vs non-severe COVID-19		MASP-1 ↑<br>MASP-2 ↑<br>MAp19 ↑	

Analysis of first samples after COVID-19 diagnosis

Complete COVID-19 cohort

Associations with higher (↑) or lower (↓) concentrations	Pattern recognition molecules	MBL associated serine proteases and proteins	Complement activity
Dialysis control vs healthy control			
Non-severe COVID-19 vs dialysis control	CL-K1 ↑	MASP-3 ↓	C3dg ↑
Severe COVID-19 vs non-severe COVID-19			

COVID-19 haemodialysis patients only

Associations with higher (↑) or lower (↓) concentrations	Pattern recognition molecules	MBL associated serine proteases and proteins	Complement activity
Dialysis control vs healthy control			
Non-severe COVID-19 vs dialysis control	CL-K1 ↑	MASP-3 ↓	
Severe COVID-19 vs non-severe COVID-19			

Supplementary Figure 5: Summary of associations between lectin pathway protein concentrations and COVID-19 in the complete study cohort and in only COVID-19 patients established on chronic haemodialysis at enrolment. Associations reached statistical significance ($p<0.05$) after adjustment for multiple analyses

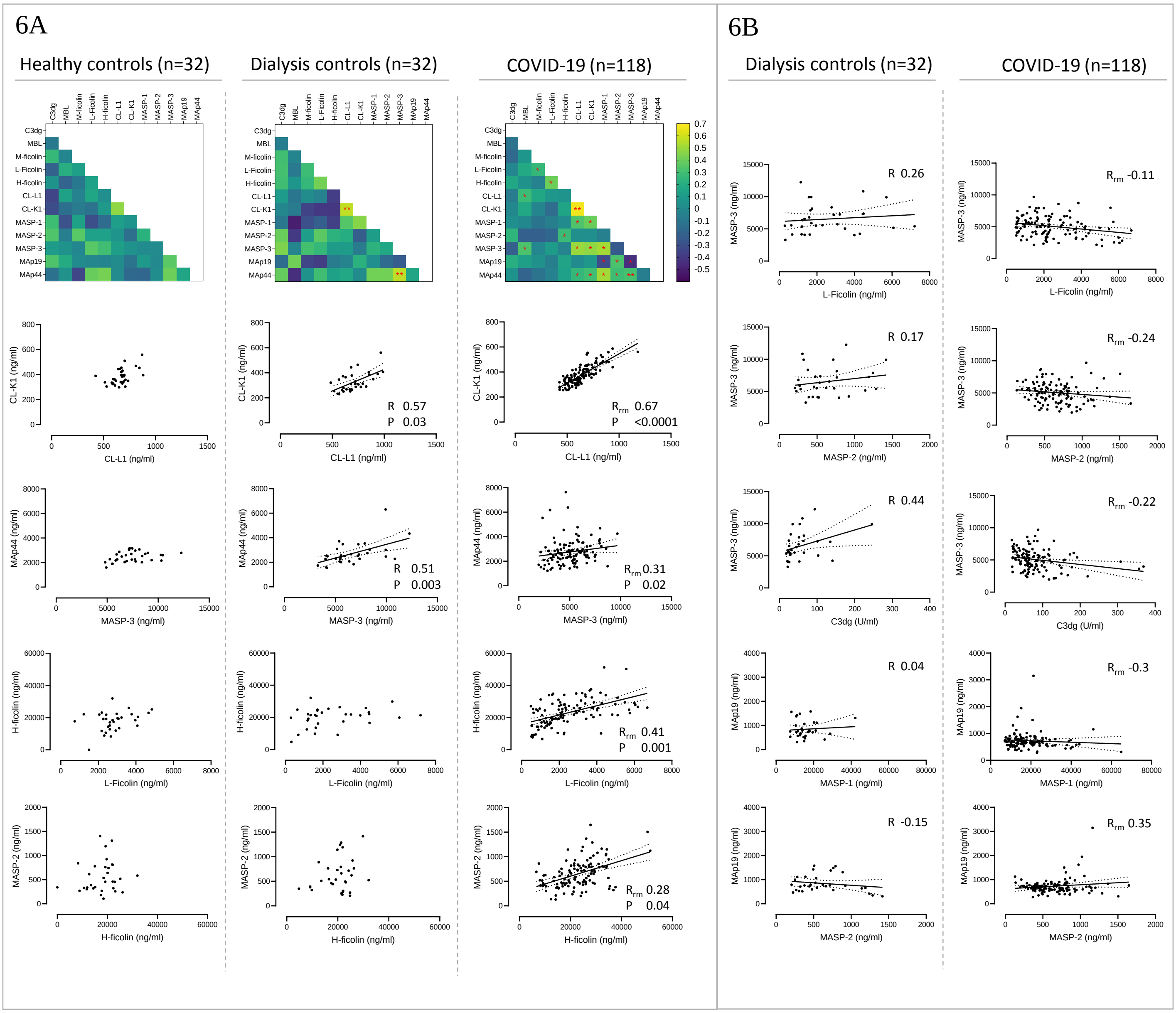

Supplementary Figure 6: Correlations between lectin pathway proteins are influenced by COVID-19.

(A) A selection of correlation plots from the Figure 4D heat maps of lectin complement protein levels. We measured lectin pathway protein concentrations from 32 samples from 32 healthy control individuals, 32 samples from 32 haemodialysis patients without COVID-19 (dialysis controls) and 118 samples from 33 patients with kidney impairment and COVID-19. We detected 20 correlations in the COVID-19 cohort, two in the dialysis controls cohort and zero in the healthy controls cohort that reached statistical significance after adjusting p-values for multiple comparisons. However, for most lectin proteins, the correlation plot appeared similar in each cohort. Four examples are shown. This suggested the differences in significant correlations between cohorts were caused by the different number of samples available for each cohort. (B) However, correlations between MASP-3, MAp19 and other lectin complement proteins adopted different correlation patterns in the COVID-19 cohort (right-hand column) compared to the dialysis controls cohort (left-hand column).

Each dot represents one sample and lines of best fit (solid line) and 95% confidence intervals (95% CI) are shown. For the healthy controls and dialysis controls cohorts, we calculated Pearson correlations (R) on log-transformed data. For the COVID-19 cohort, we applied linear mixed models and repeated measures correlation technique (rmcorr) of log-transformed data to calculate correlations (R_{rm}). We adjusted p-values for multiple comparisons using the method of Benjamini and Hochberg with a false discovery rate (Q) of 5%.

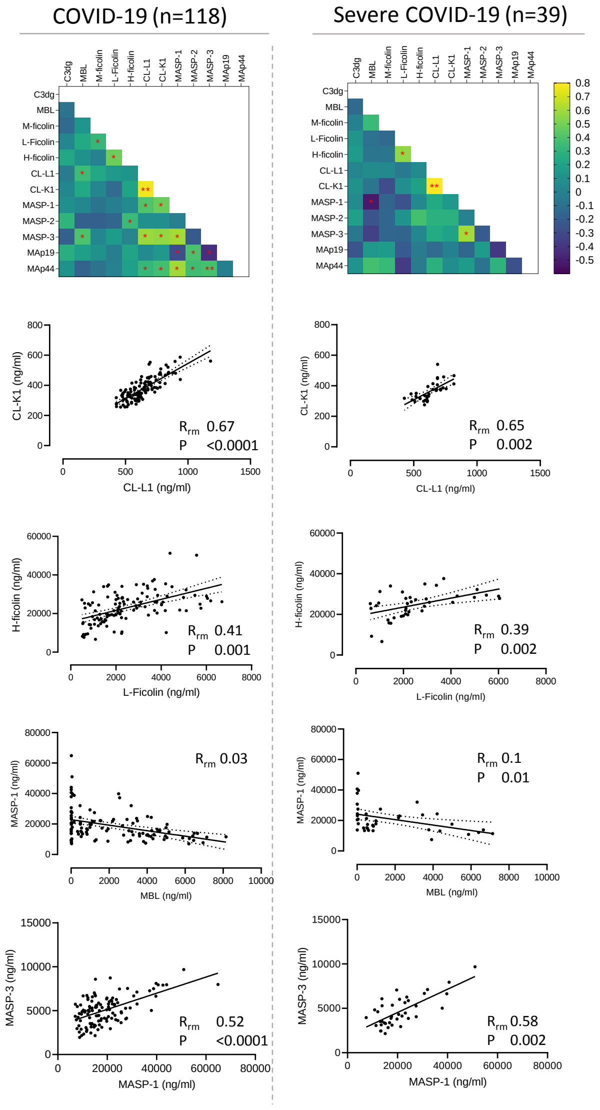

Supplementary Figure 7: Correlations between lectin pathway proteins are similar in severe COVID-19 as the total COVID-19 cohort.

The Severe COVID-19 column shows analyses of the 39 COVID-19 samples taken from cases with severe COVID-19. The COVID-19 column shows similar data to Supplementary figure 6 and is included for comparison. After adjustment for multiple analyses, we identified four lectin protein correlations that reached statistical significance in the severe COVID-19 cohort. However, the direction and nature of the associations were similar to the total COVID-19 cohort, suggesting the severity of COVID-19 did not significantly alter correlations between lectin pathway proteins.

Each dot represents one sample and lines of best fit (solid line) and 95% confidence intervals (95% CI) are shown. For the healthy controls and dialysis controls cohorts, we calculated Pearson correlations (R) on log-transformed data. For the COVID-19 cohort, we applied linear mixed models and repeated measures correlation technique (rmcorr) of log-transformed data to calculate correlations (R_{rm}). We adjusted p-values for multiple comparisons using the method of Benjamini and Hochberg with a false discovery rate (Q) of 5%.

Jack Gisby †
Candice Clarke ‡ †
Nicholas R. Medjeral-Thomas ‡ †
Maria Prendecki ‡ †
Paige Mortimer †
Eleanor Sandhu ‡ †
Stephen McAdoo ‡ †
Norzawani Buang †
Shanice Lewis †
Talat Malik †
Marie Pereira †
Frederic Toulza †
Ester Fagnano †
Marie-Anne Mawhin †
Emma Dutton †
Lunnathaya Tapeng †
Jacques Behmoara †
Matthew C. Pickering †
Marina Botto †
Michelle Willicombe ‡ †
David. C. Thomas ‡ †
James E. Peters †
Anne Troldborg §
Annette G. Hansen §
Steffen Thiel §
Liz Lightstone ‡ †
Megan Griffith ‡ †
Moira Appelbe ‡
Damien R. Ashby ‡
Edwina Brown ‡
Tom Cairns ‡
Rawya Charif ‡
Marie Condon ‡
Richard W. Corbett ‡
Neill Duncan ‡
Claire Edwards ‡
Andrew Frankel ‡
Julie Harris ‡
Peter Hill ‡
Christopher Jones ‡
Andreas Kousios ‡
Marina Loucaidou ‡
Gaetano Lucisano ‡
Kathleen Lynch ‡
A Mclean ‡
Aanad Muthusamy ‡
Margaret Nevin ‡
Andrew Palmer ‡
Darren Parsons ‡
Victoria Prout ‡
Sue Punzalan ‡
Emma Salisbury ‡
Anisha Tanna ‡
James Tomlinson ‡
Jeremy B. Levy ‡

Supplementary Appendix: Clinical and research staff who have contributed to the research.

§ Department of Biomedicine, Aarhus University, Denmark.
† Centre for Inflammatory Disease, Imperial College London, United Kingdom.
‡ Renal and Transplant Centre, Hammersmith Hospital, Imperial College Healthcare NHS Trust, London, United Kingdom.
